# Supplementary material for: Persistent Sleep Quality Deterioration among Post-COVID-19 Patients: Results from a 6-Month Follow-Up Study
Source: J Pers Med. 2022 Nov 16;12(11):1909. doi: 10.3390/jpm12111909 (PMC9692708; doi:10.3390/jpm12111909)
Supplement: Supplementary file 1 [file jpm-12-01909-s001.zip › jpm-2008255-supplementary.pdf]

**Supplementary material**

**Table S1. Comparison of PSQI Global score, Epworth sleepiness scale, FSS, STOP-Bang scale, AIS, according to participants' characteristics**

| Variables | PSQI Global score | | p |
|---|---|---|---|
| | <5 | ≥5 | |
| **Gender** | | | 0.110[b] |
| Male | 14(77.8%) | 56(57.7%) | |
| Female | 4(22.2%) | 41(42.3%) | |
| **Age, Mean (SD)** | 54.4(12.89) | 56.3(11.64) | 0.528[c] |
| **BMI, Median (IQR)** | 29(25.7-31-7) | 29(26-32.5) | 0.816[a] |
| **Smoking status** | | | 0.489[b] |
| Never smoked | 7(38.9%) | 52(54.7%) | |
| Former smoker | 9(50%) | 33(34.7%) | |
| Current smoker | 2(11.1%) | 10(10.5%) | |
| **Arterial Hypertension** | | | 0.427[b] |
| Yes | 4(22.2%) | 30(31.6%) | |
| No | 14(77.8%) | 65(68.4%) | |
| **Severity** | | | 0.224[d] |
| Group 1 | 0(0%) | 10(11.1%) | |
| Group 2 | 8(50%) | 55(61.1%) | |
| Group 3 | 5(31.3%) | 15(16.7%) | |
| Group 4 | 3(18.8%) | 10(11.1%) | |
| **Charlson Comorbidity Index, Median(IQR)** | 1(1-2.3) | 2(1-3) | 0.827[a] |
| **Length of hospital stay (days), Median(IQR)** | 12(8-18) | 10(6-16) | 0.162[a] |

*a; Mann-Whitney test, b; Chi-square test, c; Independent samples t-test, d; Fischer's exact test*

| Variables | Epworth sleepiness scale | | p |
|---|---|---|---|
| | No EDS (≤10) | EDS (>10) | |
| **Gender** | | | 0.997[b] |
| Male | 63(61.2%) | 11(61.1%) | |
| Female | 40(38.8%) | 7(38.9%) | |
| **Age, Median(IQR)** | 57(51-66) | 48(42.5-56.5) | **0.002**[a] |
| **BMI, Median(IQR)** | 29(26-32) | 29(26-34) | 0.741[a] |
| **Smoking status** | | | 0.102[b] |
| Never smoked | 54(52.9%) | 14(77.8%) | |
| Former smoker | 36(35.3%) | 4(22.2%) | |
| Current smoker | 12(11.8%) | 0(0%) | |
| **Arterial Hypertension** | | | 0.823[b] |

| | | | |
|---|---|---|---|
| Yes | 31(30.4%) | 5(27.8%) | |
| No | 71(69.6%) | 13(72.2%) | |
| **Severity** | | | <0.999[c] |
| Group 1 | 9(9.4%) | 1(6.3%) | |
| Group 2 | 58(60.4%) | 10(62.5%) | |
| Group 3 | 18(18.8%) | 3(18.8%) | |
| Group 4 | 11(11.5%) | 2(12.5%) | |
| **Charlson Comorbidity Index, Median(IQR)** | 2(1-3) | 0(0-2) | **0.001**[a] |
| **Length of hospital stay (days), Median(IQR)** | 11(7-17) | 8(5.5-14) | 0.168[a] |

*a; Mann-Whitney test, b; Chi-square test, c; Fischer's exact test*

| Variables | Epworth sleepiness scale | | | | p |
|---|---|---|---|---|---|
| | **Normal Sleepiness (0-10)** | **Mild sleepiness (11-14)** | **Moderate Sleepiness (15-17)** | **Severe Sleepiness (18-24)** | |
| **Gender** | | | | | 0.098[b] |
| Male | 63(61.2%) | 9(69.2%) | 0(0%) | 2(100%) | |
| Female | 40(38.8%) | 4(30.8%) | 3(100%) | 0(0%) | |
| **Age, Median(IQR)** | 57(51-66) | 47(37.5-49.5) | 58 | 62 | **<0.001**[a] |
| **BMI, Median(IQR)** | 29(26-32) | 29(25.2-34) | 31 | 28.5 | 0.892[a] |
| **Smoking status** | | | | | 0.502[b] |
| Never smoked | 54(52.9%) | 10(76.9%) | 3(100%) | 1(50%) | |
| Former smoker | 36(35.3%) | 3(23.1%) | 0(0%) | 1(50%) | |
| Current smoker | 12(11.8%) | 0(0%) | 0(0%) | 0(0%) | |
| **Arterial Hypertension** | | | | | 0.015[b] |
| Yes | 31(30.4%) | 1(7.7%) | 2(66.7%) | 2(100%) | |
| No | 71(69.6%) | 12(92.3%) | 1(33.3%) | 0(0%) | |
| **Severity** | | | | | 0.953[c] |
| Group 1 | 9(9.4%) | 1(7.7%) | 0(0%) | 0(0%) | |
| Group 2 | 58(60.4%) | 8(61.5%) | 1(100%) | 1(50%) | |
| Group 3 | 18(18.8%) | 2(15.4%) | 0(0%) | 1(50%) | |
| Group 4 | 11(11.5%) | 2(15.4%) | 0(0%) | 0(0%) | |
| **Charlson Comorbidity Index, Median(IQR)** | 2(1-3) | 0(0-0.5) | 2(0-0) | 2(2-2) | **<0.001**[a] |
| **Length of hospital stay (days), Median(IQR)** | 11(7-17) | 8(5.5-14) | 6.5(4-0) | 19.5(6-0) | 0.196[a] |

*a; Mann-Whitney test, b; Chi-square test, c; Fischer's exact test*

| Variables | FSS <4 | FSS ≥4 | p |
|---|---|---|---|
| **Gender** | | | 0.474[b] |
| Male | 38(64.4%) | 36(58.1%) | |
| Female | 21(35.6%) | 26(41.9%) | |
| **Age, Mean(SD)** | 59.1(10.41) | 53(12.03) | **0.004**[a] |
| **BMI, Mean(SD)** | 28.6(4.58) | 30.5(5.50) | **0.043**[a] |
| **Smoking status** | | | 0.705[b] |
| Never smoked | 34(57.6%) | 34(55.7%) | |
| Former smoker | 18(30.5%) | 22(36.1%) | |
| Current smoker | 7(11.9%) | 5(8.2%) | |
| **Arterial Hypertension** | | | 0.604[b] |
| Yes | 19(32.2%) | 17(27.9%) | |
| No | 40(67.8%) | 44(72.1%) | |
| **Severity** | | | 0.928[b] |
| Group 1 | 5(9.1%) | 5(8.8%) | |
| Group 2 | 35(63.6%) | 33(57.9%) | |
| Group 3 | 9(16.4%) | 12(21.1%) | |
| Group 4 | 6(10.9%) | 7(12.3%) | |
| **Charlson Comorbidity Index, Median(IQR)** | 2(1-3) | 1(0-2) | **0.042**[c] |
| **Length of hospital stay (days), Median(IQR)** | 11(7-16.5) | 9(6-16.8) | 0.509[c] |

*a; Independent samples t-test, b; Chi-square test, c; Mann-Whitney test*

| Variables | STOP-Bang Low risk (≤2) | Intermediate risk (3-4) | High risk (≥5) | p |
|---|---|---|---|---|
| **Gender** | | | | **<0.001**[b] |
| Male | 22(40.7%) | 38(74.5%) | 14(87.5%) | |
| Female | 32(59.3%) | 13(25.5%) | 2(12.5%) | |
| **Age, Mean(SD)** | 54.5(11.80) | 58.2(11.55) | 53.9(10.78) | 0.202[a] |
| **BMI, Median(IQR)** | 27(25-31) | 30(26-32) | 33(29-35) | **0.004**[c] |
| **Smoking status** | | | | 0.391[b] |
| Never smoked | 35(64.8%) | 25(50%) | 8(50%) | |
| Former smoker | 13(24.1%) | 21(42%) | 6(37.5%) | |
| Current smoker | 6(11.1%) | 4(8%) | 2(12.5%) | |
| **Arterial Hypertension** | | | | **<0.001**[b] |
| Yes | 7(13%) | 20(40%) | 9(56.3%) | |
| No | 47(87%) | 30(60%) | 7(43.8%) | |
| **Severity** | | | | 0.707[d] |
| Group 1 | 3(6.1%) | 6(12.5%) | 1(6.7%) | |
| Group 2 | 30(61.2%) | 28(58.3%) | 10(66.7%) | |

| | | | | |
|---|---|---|---|---|
| Group 3 | 9(18.4%) | 8(16.7%) | 4(26.7%) | |
| Group 4 | 7(14.3%) | 6(12.5%) | 0(0%) | |
| **Charlson Comorbidity Index, Median(IQR)** | 1(0-2) | 2(1-3.3) | 1.5(1-2) | 0.113[c] |
| **Length of hospital stay (days), Median(IQR)** | 9(6-16.8) | 12(6.5-16) | 8.5(7-24) | 0.780[c] |

*a; One-way ANOVA, b; Chi-square test, c; Kruskal Wallis test, d; Fischer's exact test*

| | Athens Insomnia Scale | | |
|---|---|---|---|
| **Variables** | **<6** | **≥6** | **p** |
| **Gender** | | | **<0.001**[b] |
| Male | 43(79.6%) | 31(44.3%) | |
| Female | 11(20.4%) | 39(55.7%) | |
| **Age, Mean(SD)** | 57.8(11.71) | 54.7(11.53) | 0.138[a] |
| **BMI, Mean(SD)** | 28.5(3.72) | 30.6(6.02) | **0.018**[a] |
| **Smoking status** | | | 0.852[b] |
| Never smoked | 28(52.8%) | 40(58%) | |
| Former smoker | 19(35.8%) | 22(31.9%) | |
| Current smoker | 6(11.3%) | 7(10.1%) | |
| **Arterial Hypertension** | | | 0.846[b] |
| Yes | 17(32.1%) | 21(30.4%) | |
| No | 36(67.9%) | 48(69.6%) | |
| **Severity** | | | 0.512[b] |
| Group 1 | 3(6%) | 7(10.9%) | |
| Group 2 | 30(60%) | 40(62.5%) | |
| Group 3 | 12(24%) | 9(14.1%) | |
| Group 4 | 5(10%) | 8(12.5%) | |
| **Charlson Comorbidity Index, Median(IQR)** | 2(1-3) | 1(0.8-3) | 0.204[c] |
| **Length of hospital stay (days), Median(IQR)** | 12(8-18) | 8(5.3-15.8) | **0.014**[c] |

*a; Independent samples t-test, b; Chi-square test, c; Mann-Whitney test*

| | Athens Insomnia Scale | | | | |
|---|---|---|---|---|---|
| **Variables** | **Absence of insomnia (0–5)** | **Mild (6-9)** | **Moderate (10-15)** | **Severe (16-24)** | **p** |
| **Gender** | | | | | **<0.001**[b] |
| Male | 43(79.6%) | 11(36.7%) | 16(53.3%) | 4(40%) | |
| Female | 11(20.4%) | 19(63.3%) | 14(46.7%) | 6(60%) | |
| **Age, Mean(SD)** | 57.81(11.71) | 56.20(10.52) | 52.86(11.23) | 55.3(15.41) | 0.331[a] |
| **BMI, Mean(SD)** | 28.49(3.72) | 29.86(6.15) | 30.93(5.6) | 32(7.12) | 0.092[a] |
| **Smoking status** | | | | | 0.778[c] |
| Never smoked | 28(52.8%) | 15(50%) | 19(65.5%) | 6(60%) | |

| | | | | | |
|---|---|---|---|---|---|
| Former smoker | 19(35.8%) | 10(33.3%) | 8(27.6%) | 4(40%) | |
| Current smoker | 6(11.3%) | 5(16.7%) | 2(6.9%) | 0(0%) | |
| **Arterial Hypertension** | | | | | 0.773[b] |
| Yes | 17(32.1%) | 10(33.3%) | 7(24.1%) | 4(40%) | |
| No | 36(67.9%) | 20(66.7%) | 22(75.9%) | 6(60%) | |
| **Severity** | | | | | 0.824[c] |
| Group 1 | 3(6%) | 4(13.3%) | 3(11.5%) | 0(0%) | |
| Group2 | 30(60%) | 17(56.7%) | 16(61.5%) | 7(87.5%) | |
| Group 3 | 12(24%) | 5(16.7%) | 4(15.4%) | 0(0%) | |
| Group 4 | 5(10%) | 4(13.3%) | 3(11.5%) | 1(12.5%) | |
| **Charlson Comorbidity Index, Median(IQR)** | 2(1-3) | 2(1-2.3) | 1(0-3) | 1.5(0-3) | 0.471[d] |
| **Length of hospital stay (days), Median(IQR)** | 12(8-18) | 8(5-15.5) | 8(6-16.5) | 8(4.8-13) | 0.091[d] |

*a; One-way ANOVA, b; Chi-square test, c; Fischer's exact test, d; Kruskal-Wallis test*

**Table S2. Normality tests for Charlson comorbidity index and Length of hospital stay according to PSQI Global score, Epworth sleepiness scale, FSS, STOP-Bang scale, AIS**

| | | Tests of Normality | | |
|---|---|---|---|---|
| | | Kolmogorov-Smirnov/ Shapiro-Wilk | | |
| | PSQI Global score | Statistic | df | p |
| Charlson comorbidity index | <5 | 0.875 | 18 | 0.021 |
| | ≥5 | 0.181 | 96 | <0.001 |
| Length of hospital stay (days) | <5 | 0.845 | 17 | 0.009 |
| | ≥5 | 0.188 | 95 | <0.001 |

| | | Tests of Normality | | |
|---|---|---|---|---|
| | | Kolmogorov-Smirnov/ Shapiro-Wilk | | |
| | Epworth sleepiness scale | Statistic | df | p |
| Charlson comorbidity index | No EDS (≤10) | 0.201 | 102 | <0.001 |
| | EDS (>10) | 0.717 | 18 | <0.001 |
| Length of hospital stay (days) | No EDS (≤10) | 0.146 | 100 | <0.001 |
| | EDS (>10) | 0.813 | 17 | 0.003 |

| | | Tests of Normality | | |
|---|---|---|---|---|
| | | Kolmogorov-Smirnov/ Shapiro-Wilk | | |

| | Epworth sleepiness scale | Statistic | df | p |
|---|---|---|---|---|
| Charlson comorbidity index | Normal Sleepiness (0-10) | 0.201 | 102 | <0.001 |
| | Mild sleepiness (11-14) | 0.456 | 13 | <0.001 |
| | Moderate Sleepiness (15-17) | 0.964 | 3 | 0.637 |
| | Severe Sleepiness (18-24) | | 2 | |
| Length of hospital stay (days) | Normal Sleepiness (0-10) | 0.146 | 100 | <0.001 |
| | Mild sleepiness (11-14) | 0.146 | 13 | .200* |
| | Moderate Sleepiness (15-17) | 0.260 | 2 | |
| | Severe Sleepiness (18-24) | 0.260 | 2 | |

| | | **Tests of Normality** | | |
|---|---|---|---|---|
| | | Kolmogorov-Smirnov/ Shapiro-Wilk | | |
| | FSS | Statistic | df | p |
| Charlson comorbidity index | <4 | 0.213 | 59 | <0.001 |
| | ≥4 | 0.197 | 61 | <0.001 |
| Length of hospital stay (days) | <4 | 0.163 | 57 | 0.001 |
| | ≥4 | 0.172 | 60 | <0.001 |

| | | **Tests of Normality** | | |
|---|---|---|---|---|
| | | Kolmogorov-Smirnov/ Shapiro-Wilk | | |
| | STOP-Bang | Statistic | df | p |
| Charlson comorbidity index | Low risk (≤2) | 0.191 | 54 | <0.001 |
| | Intermediate risk (3-4) | 0.201 | 50 | <0.001 |
| | High risk (≥5) | 0.897 | 16 | 0.071 |
| Length of hospital stay (days) | Low risk (≤2) | 0.192 | 52 | <0.001 |
| | Intermediate risk (3-4) | 0.112 | 49 | 0.169 |
| | High risk (≥5) | 0.787 | 16 | 0.002 |

| | | **Tests of Normality** | | |
|---|---|---|---|---|
| | | Kolmogorov-Smirnov/ Shapiro-Wilk | | |
| | Athens Insomnia Scale | Statistic | df | p |
| Charlson comorbidity index | <6 | 0.217 | 53 | <0.001 |
| | ≥6 | 0.193 | 70 | <0.001 |
| Length of hospital stay (days) | <6 | 0.177 | 52 | <0.001 |
| | ≥6 | 0.179 | 68 | <0.001 |

| | | **Tests of Normality** | | |
|---|---|---|---|---|

| | Athens Insomnia Scale | Kolmogorov-Smirnov/ Shapiro-Wilk Statistic | df | p |
|---|---|---|---|---|
| Charlson comorbidity index | Absence of insomnia (0–5) | 0.217 | 53 | <0.001 |
| | Mild (6-9) | 0.197 | 30 | 0.004 |
| | Moderate (10-15) | 0.227 | 30 | <0.001 |
| | Severe (16-24) | 0.852 | 10 | 0.061 |
| Length of hospital stay (days) | Absence of insomnia (0–5) | 0.177 | 52 | <0.001 |
| | Mild (6-9) | 0.162 | 29 | 0.049 |
| | Moderate (10-15) | 0.244 | 29 | <0.001 |
| | Severe (16-24) | 0.869 | 10 | 0.096 |

**Table S3. Mixed effects model for assessment tools**

| | **b** | **SE** | **t** | **p** | **95% CI** |
|---|---|---|---|---|---|
| ***Epworth Sleepiness Scale*** | | | | | |
| Time | -0.138 | 0.183 | -0.75 | 0.452 | (-0.497,0.222) |
| Gender | | | | | |
| Male | 1.092 | 0.691 | 1.58 | 0.114 | (-0.264,2.447) |
| Female | Ref. category | | | | |
| Age | -0.065 | 0.047 | -1.37 | 0.171 | (-0.157,0.028) |
| BMI | 0.084 | 0.065 | 1.28 | 0.199 | (-0.044,0.211) |
| Severity | | | | | |
| Group 1 | Ref. category | | | | |
| Group 2 | -1.19 | 1.173 | -1.01 | 0.310 | (-3.490,1.110) |
| Group 3 | -2.165 | 1.33 | -1.63 | 0.103 | (-4.771,0.441) |
| Group 4 | -2.264 | 1.447 | -1.56 | 0.118 | (-5.100,0.572) |
| Charlson Comorbidity Index | -0.398 | 0.34 | -1.17 | 0.242 | (-1.064,0.268) |
| ***FSS Scale*** | | | | | |
| Time | -0.376 | 0.086 | -4.37 | **<0.001** | (-0.544,-0.207) |
| Gender | | | | | |
| Male | -0.398 | 0.289 | -1.38 | 0.169 | (-0.965,0.169) |

| | | | | | |
|---|---|---|---|---|---|
| Female | Ref. category | | | | |
| Age | -0.04 | 0.02 | -2.02 | 0.043 | (-0.079,-0.001) |
| BMI | 0.067 | 0.027 | 2.46 | 0.014 | (0.014,0.121) |
| Severity | | | | | |
| Group 1 | Ref. category | | | | |
| Group 2 | -0.152 | 0.49 | -0.31 | 0.757 | (-1.112,0.808) |
| Group 3 | -0.118 | 0.555 | -0.21 | 0.832 | (-1.206,0.970) |
| Group 4 | -0.355 | 0.604 | -0.59 | 0.557 | (-1.539,0.830) |
| Charlson Comorbidity Index | 0.167 | 0.142 | 1.18 | 0.240 | (-0.111,0.445) |
| ***STOP-Bang*** | | | | | |
| Time | 0.022 | 0.058 | 0.38 | 0.704 | (-0.092,0.136) |
| Gender | | | | | |
| Male | 1.268 | 0.224 | 5.67 | <0.001 | (0.830,1.706) |
| Female | Ref. category | | | | |
| Age | 0.02 | 0.015 | 1.28 | 0.200 | (-0.010,0.050) |
| BMI | 0.097 | 0.021 | 4.59 | <0.001 | (0.055,0.138) |
| Severity | | | | | |
| Group 1 | Ref. category | | | | |
| Group 2 | -0.155 | 0.379 | -0.41 | 0.683 | (-0.899,0.589) |
| Group 3 | -0.163 | 0.43 | -0.38 | 0.704 | (-1.006,0.679) |
| Group 4 | -0.684 | 0.468 | -1.46 | 0.144 | (-1.601,0.233) |
| Charlson Comorbidity Index | 0.032 | 0.11 | 0.29 | 0.770 | (-0.183,0.248) |
| ***Athens Insomnia Scale*** | | | | | |
| Time | -0.487 | 0.244 | -2 | **0.046** | (-0.964,-0.009) |
| Gender | | | | | |
| Male | -2.775 | 0.919 | -3.02 | 0.003 | (-4.577,-0.973) |
| Female | Ref. category | | | | |
| Age | -0.034 | 0.063 | -0.55 | 0.585 | (-0.157,0.089) |
| BMI | 0.178 | 0.087 | 2.05 | 0.040 | (0.008,0.348) |

| | | | | | |
|---|---|---|---|---|---|
| Severity | | | | | |
| Group 1 | Ref. category | | | | |
| Group 2 | -0.991 | 1.592 | -0.62 | 0.534 | (-4.111,2.129) |
| Group 3 | -0.685 | 1.79 | -0.38 | 0.702 | (-4.194,2.824) |
| Group 4 | -0.926 | 1.952 | -0.47 | 0.635 | (-4.752,2.900) |
| Charlson Comorbidity Index | 0.002 | 0.454 | 0 | 0.997 | (-0.888,0.892) |

**Table S4. Mixed effects model for PSQI components and Global score**

| | b | SE | t | p | 95% CI |
|---|---|---|---|---|---|
| ***PSQI C1—Subjective sleep quality*** | | | | | |
| Time | -0.028 | 0.04 | -0.7 | 0.460 | (-0.105,0.048) |
| Gender | | | | | |
| Male | 0.017 | 0.13 | 0.13 | 0.900 | (-0.235,0.269) |
| Female | Ref. category | | | | |
| Age | -0.004 | 0.01 | -0.4 | 0.670 | (-0.021,0.014) |
| BMI | 0.024 | 0.01 | 1.96 | 0.050 | (0,0.048) |
| Severity | | | | | |
| Group 1 | Ref. category | | | | |
| Group 2 | -0.315 | 0.22 | -1.4 | 0.150 | (-0.748,0.118) |
| Group 3 | -0.44 | 0.25 | -1.8 | 0.070 | (-0.924,0.044) |
| Group 4 | -0.065 | 0.27 | -0.2 | 0.810 | (-0.590,0.460) |
| Charlson Comorbidity Index | -0.015 | 0.06 | -0.2 | 0.810 | (-0.138,0.108) |
| ***PSQI C2—Sleep latency*** | b | SE | t | P | 95% CI |
| Time | -0.083 | 0.05 | -1.8 | 0.070 | (-0.172,0.006) |
| Gender | | | | | |
| Male | -0.381 | 0.14 | -2.7 | 0.010 | (-0.654,-0.109) |
| Female | Ref. category | | | | |
| Age | 0 | 0.01 | -0.1 | 0.960 | (-0.019,0.018) |
| BMI | 0.007 | 0.01 | 0.54 | 0.590 | (-0.019,0.033) |

| | | | | | |
|---|---|---|---|---|---|
| Severity | | | | | |
| Group 1 | Ref. category | | | | |
| Group 2 | -0.189 | 0.24 | -0.8 | 0.430 | (-0.655,0.277) |
| Group 3 | -0.387 | 0.27 | -1.5 | 0.150 | (-0.908,0.134) |
| Group 4 | 0.03 | 0.29 | 0.1 | 0.920 | (-0.535,0.596) |
| Charlson Comorbidity Index | 0.036 | 0.07 | 0.53 | 0.600 | (-0.097,0.168) |
| ***PSQI C3—Sleep duration*** | b | SE | t | p | 95% CI |
| Time | 0.035 | 0.05 | 0.69 | 0.490 | (-0.064,0.133) |
| Gender | | | | | |
| Male | 0.104 | 0.16 | 0.64 | 0.530 | (-0.216,0.424) |
| Female | Ref. category | | | | |
| Age | 0.014 | 0.01 | 1.27 | 0.210 | (-0.008,0.036) |
| BMI | 0.012 | 0.02 | 0.81 | 0.420 | (-0.018,0.043) |
| Severity | | | | | |
| Group 1 | Ref. category | | | | |
| Group 2 | -0.341 | 0.28 | -1.2 | 0.220 | (-0.889,0.208) |
| Group 3 | -0.423 | 0.31 | -1.4 | 0.180 | (-1.036,0.190) |
| Group 4 | -0.395 | 0.34 | -1.2 | 0.240 | (-1.061,0.270) |
| Charlson Comorbidity Index | -0.102 | 0.08 | -1.3 | 0.200 | (-0.258,0.053) |
| ***PSQI C4—Habitual sleep efficiency*** | b | SE | t | p | 95% CI |
| Time | -0.038 | 0.08 | -0.5 | 0.630 | (-0.191,0.115) |
| Gender | | | | | |
| Male | -0.385 | 0.18 | -2.2 | 0.030 | (-0.731,-0.038) |
| Female | Ref. category | | | | |
| Age | 0.01 | 0.01 | 0.81 | 0.420 | (-0.014,0.033) |
| BMI | 0.011 | 0.02 | 0.66 | 0.510 | (-0.022,0.044) |
| Severity | | | | | |
| Group 1 | Ref. category | | | | |
| Group 2 | -0.418 | 0.3 | -1.4 | 0.160 | (-1.001,0.165) |

| | | | | | |
|---|---|---|---|---|---|
| Group 3 | -0.768 | 0.33 | -2.3 | 0.020 | (-1.422,-0.113) |
| Group 4 | -0.722 | 0.36 | -2 | 0.050 | (-1.430,-0.013) |
| Charlson Comorbidity Index | 0.021 | 0.09 | 0.24 | 0.810 | (-0.146,0.187) |
| ***PSQI C5—Sleep disturbance*** | b | SE | t | p | 95% CI |
| Time | -0.122 | 0.04 | -3.3 | **<0.001** | (-0.194,-0.05) |
| Gender | | | | | |
| Male | -0.283 | 0.1 | -2.9 | <0.001 | (-0.477,-0.089) |
| Female | Ref. category | | | | |
| Age | -0.008 | 0.01 | -1.2 | 0.230 | (-0.021,0.005) |
| BMI | 0.018 | 0.01 | 1.88 | 0.060 | (-0.001,0.036) |
| Severity | | | | | |
| Group 1 | Ref. category | | | | |
| Group 2 | -0.056 | 0.17 | -0.3 | 0.740 | (-0.386,0.273) |
| Group 3 | -0.176 | 0.19 | -0.9 | 0.350 | (-0.546,0.193) |
| Group 4 | -0.045 | 0.2 | -0.2 | 0.830 | (-0.446,0.355) |
| Charlson Comorbidity Index | 0.035 | 0.05 | 0.73 | 0.470 | (-0.059,0.129) |
| ***PSQI C6—Use of sleep medication*** | b | SE | t | p | 95% CI |
| Time | -0.033 | 0.04 | -0.8 | 0.410 | (-0.113,0.046) |
| Gender | | | | | |
| Male | -0.214 | 0.16 | -1.4 | 0.170 | (-0.517,0.090) |
| Female | Ref. category | | | | |
| Age | -0.017 | 0.01 | -1.6 | 0.110 | (-0.038,0.004) |
| BMI | -0.017 | 0.02 | -1.2 | 0.240 | (-0.046,0.011) |
| Severity | | | | | |
| Group 1 | Ref. category | | | | |
| Group 2 | -0.409 | 0.27 | -1.5 | 0.130 | (-0.931,0.113) |
| Group 3 | -0.418 | 0.3 | -1.4 | 0.160 | (-1.001,0.165) |
| Group 4 | -0.493 | 0.32 | -1.5 | 0.130 | (-1.127,0.140) |
| Charlson Comorbidity Index | 0.177 | 0.08 | 2.34 | 0.020 | (0.029,0.325) |

| ***PSQI C7—Day Time dysfunctions*** | b | SE | t | p | 95% CI |
|---|---|---|---|---|---|
| Time | -0.16 | 0.04 | -3.6 | **<0.001** | (-0.247,-0.073) |
| Gender | | | | | |
| Male | -0.213 | 0.11 | -1.9 | 0.050 | (-0.429,0.004) |
| Female | Ref. category | | | | |
| Age | -0.01 | 0.01 | -1.4 | 0.170 | (-0.025,0.004) |
| BMI | 0.012 | 0.01 | 1.17 | 0.240 | (-0.008,0.033) |
| Severity | | | | | |
| Group 1 | Ref. category | | | | |
| Group 2 | -0.005 | 0.19 | -0 | 0.980 | (-0.371,0.361) |
| Group 3 | 0.082 | 0.21 | 0.39 | 0.70 | (-0.329,0.493) |
| Group 4 | -0.296 | 0.23 | -1.3 | 0.190 | (-0.741,0.149) |
| Charlson Comorbidity Index | -0.002 | 0.05 | -0 | 0.970 | (-0.106,0.102) |
| ***PSQI—Global score*** | b | SE | t | p | 95% CI |
| Time | -0.427 | 0.17 | -2.57 | **0.010** | (-0.754,-0.101) |
| Gender | | | | | |
| Male | -1.28 | 0.65 | -1.98 | 0.048 | (-2.547,-0.014) |
| Female | Ref. category | | | | |
| Age | -0.02 | 0.05 | -0.45 | 0.653 | (-0.108,0.067) |
| BMI | 0.07 | 0.06 | 1.16 | 0.247 | (-0.049,0.190) |
| Severity | | | | | |
| Group 1 | Ref. category | | | | |
| Group 2 | -1.618 | 1.11 | -1.45 | 0.146 | (-3.800,0.564) |
| Group 3 | -2.579 | 1.24 | -2.07 | 0.038 | (-5.016,-0.142) |
| Group 4 | -1.92 | 1.35 | -1.42 | 0.155 | (-4.567,0.728) |

| | | | | | |
|---|---|---|---|---|---|
| Charlson Comorbidity Index | 0.163 | 0.32 | 0.52 | 0.605 | (-0.456,0.782) |

**Figures S1-S12 (ANOVA illustrated results)**

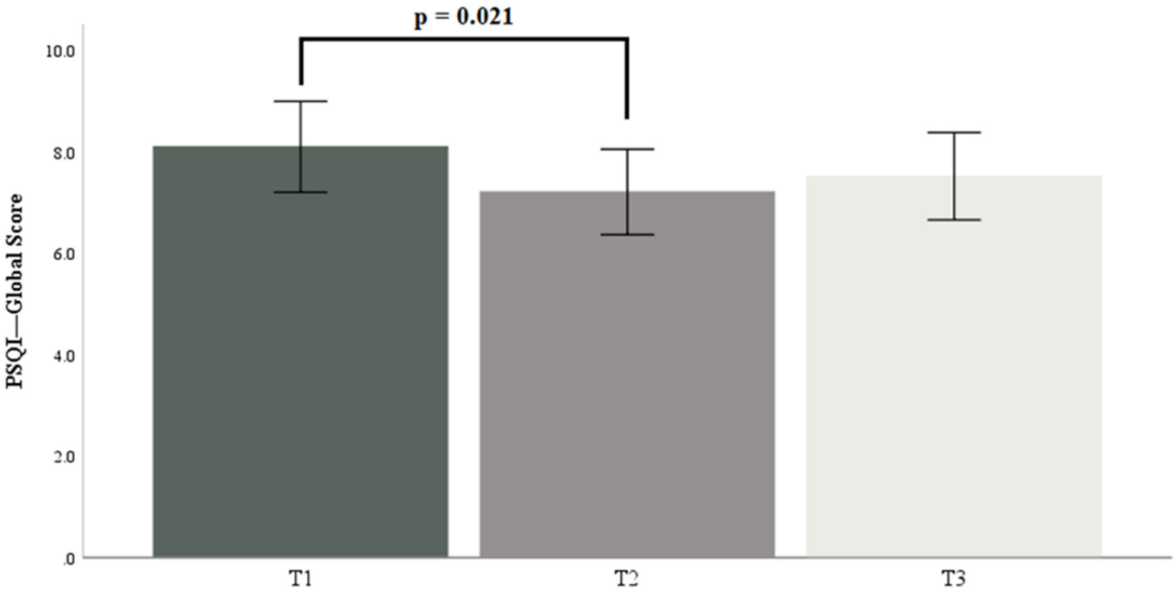


**Figure S1.** Mean PSQI—Global score for the three time points

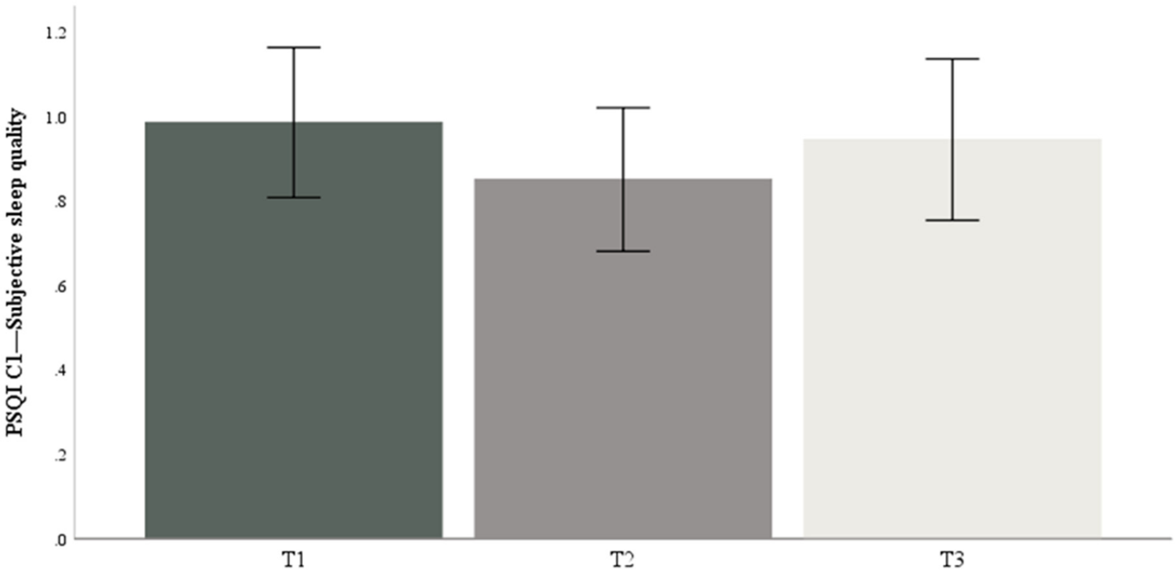


**Figure S2.** Mean PSQI C1—Subjective sleep quality score for the three time points

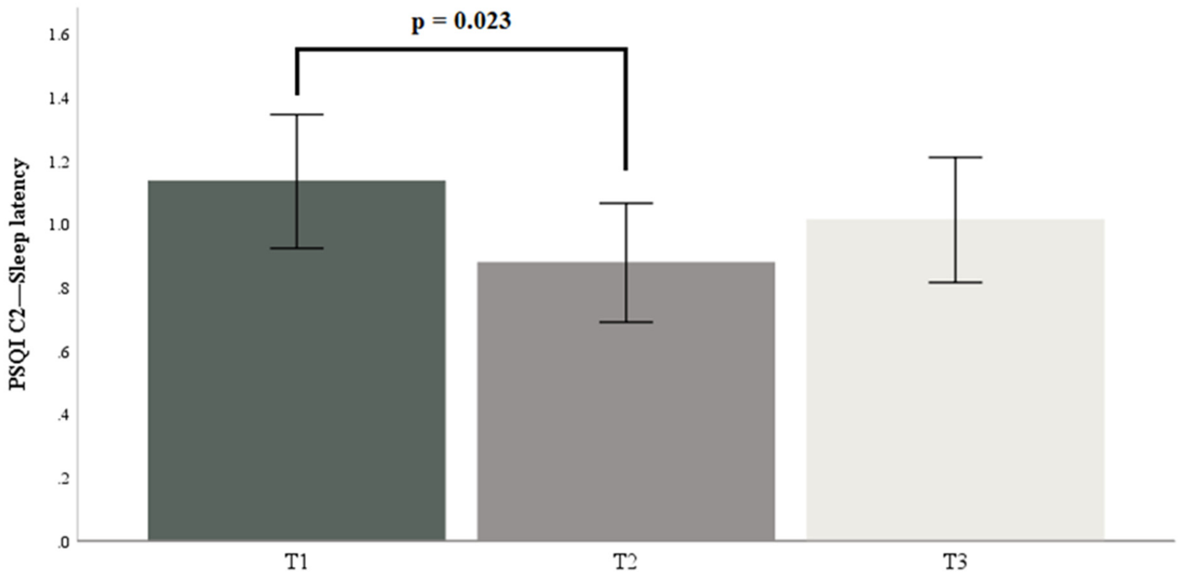


**Figure S3.** Mean PSQI C2—Sleep latency score for the three time points

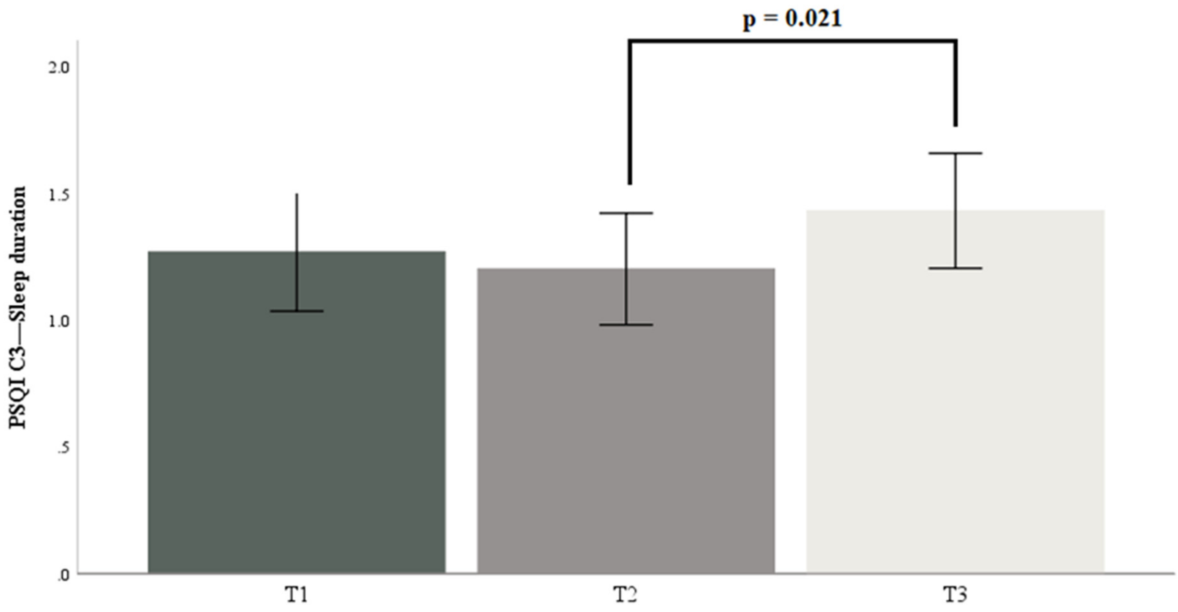


**Figure S4.** Mean PSQI C3—Sleep duration score for the three time points

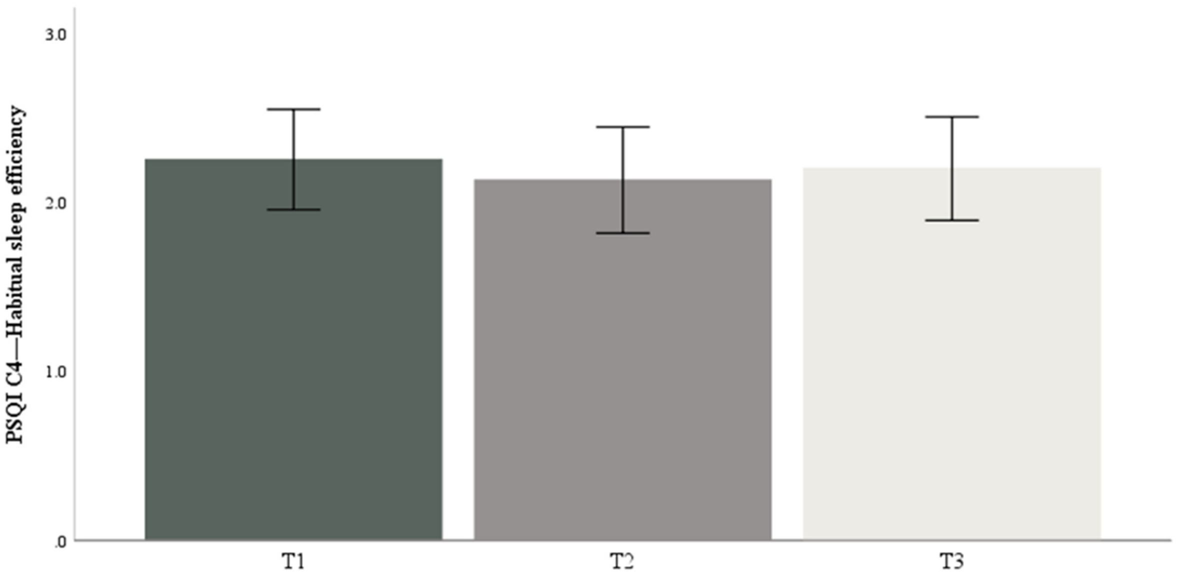


**Figure S5.** Mean PSQI C4—Habitual sleep efficiency score for the three time points

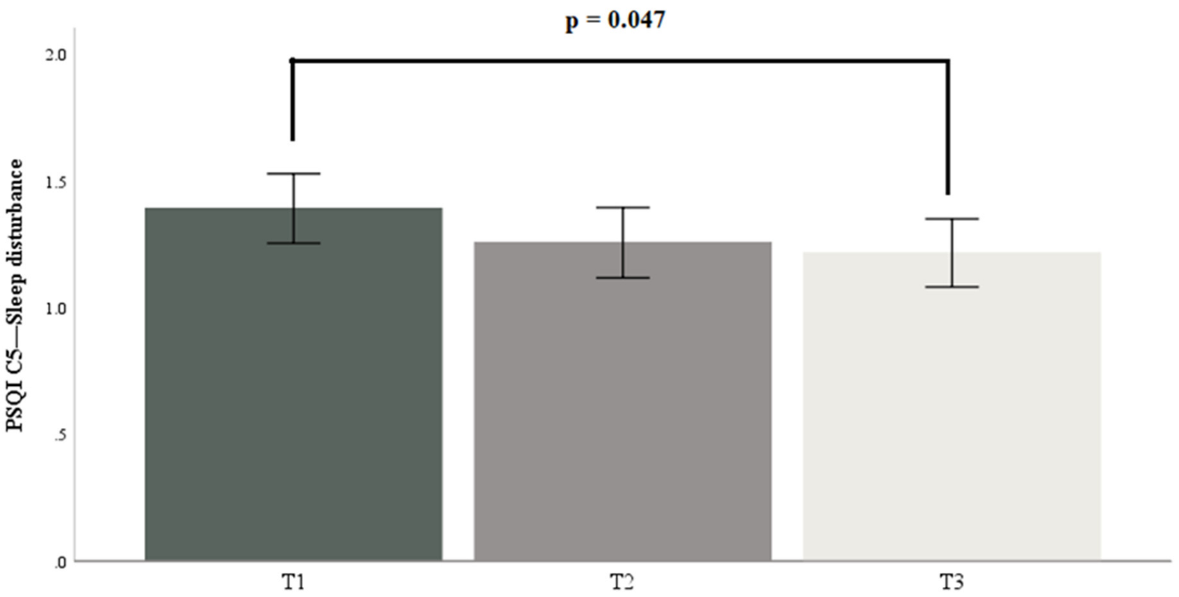


**Figure S6.** Mean PSQI C5—Sleep disturbance score for the three time points

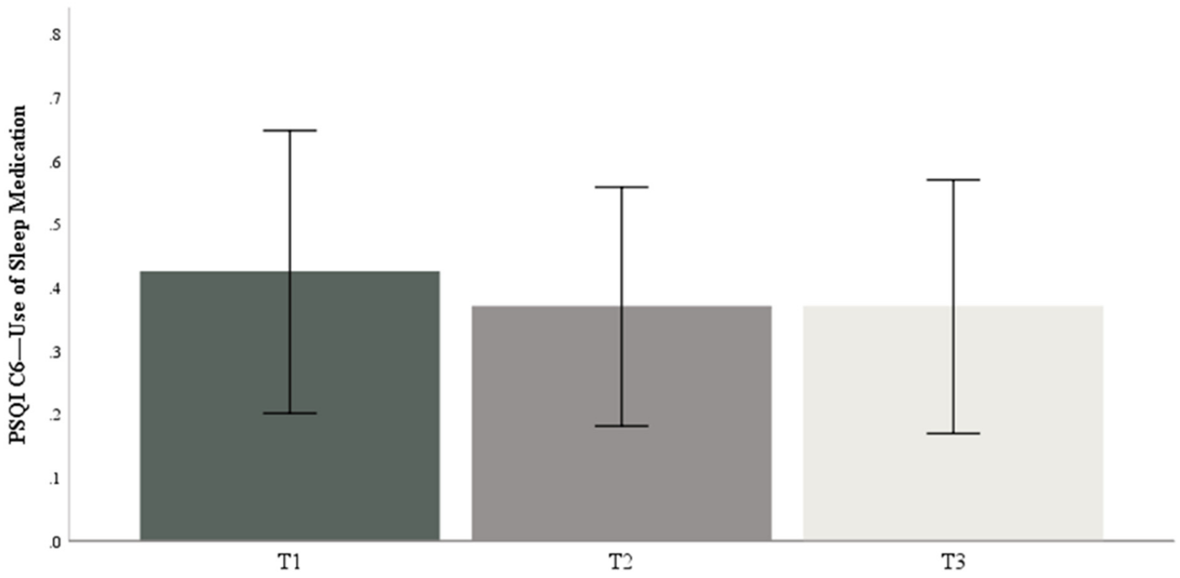


**Figure S7.** Mean PSQI C6—Use of Sleep Medication score for the three time points

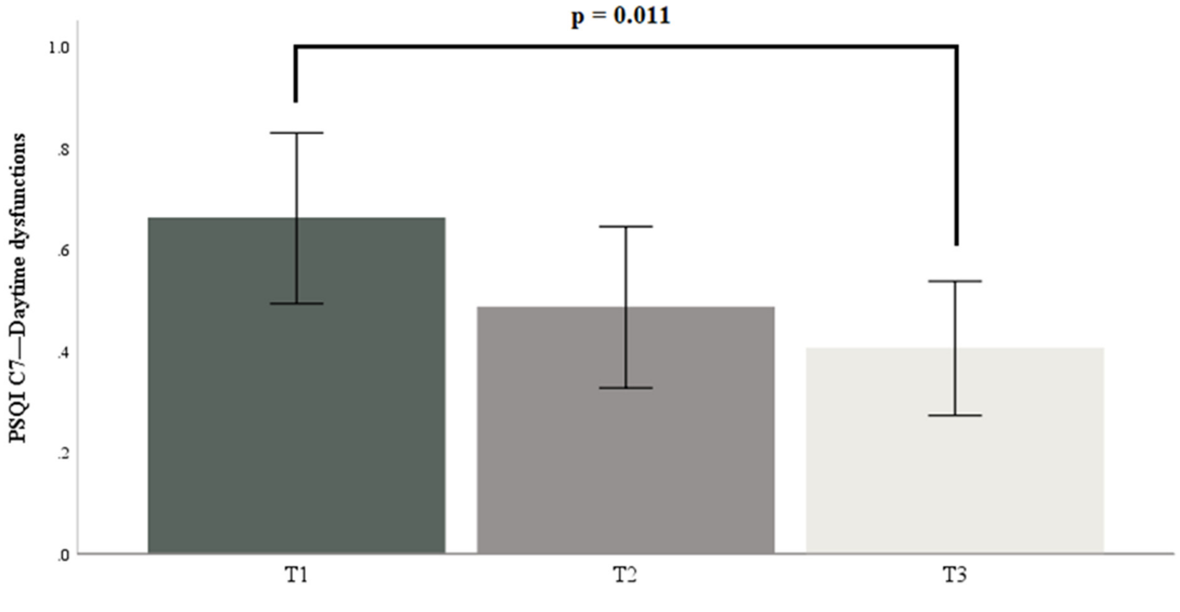


**Figure S8.** Mean PSQI C7—Daytime dysfunctions score for the three time points

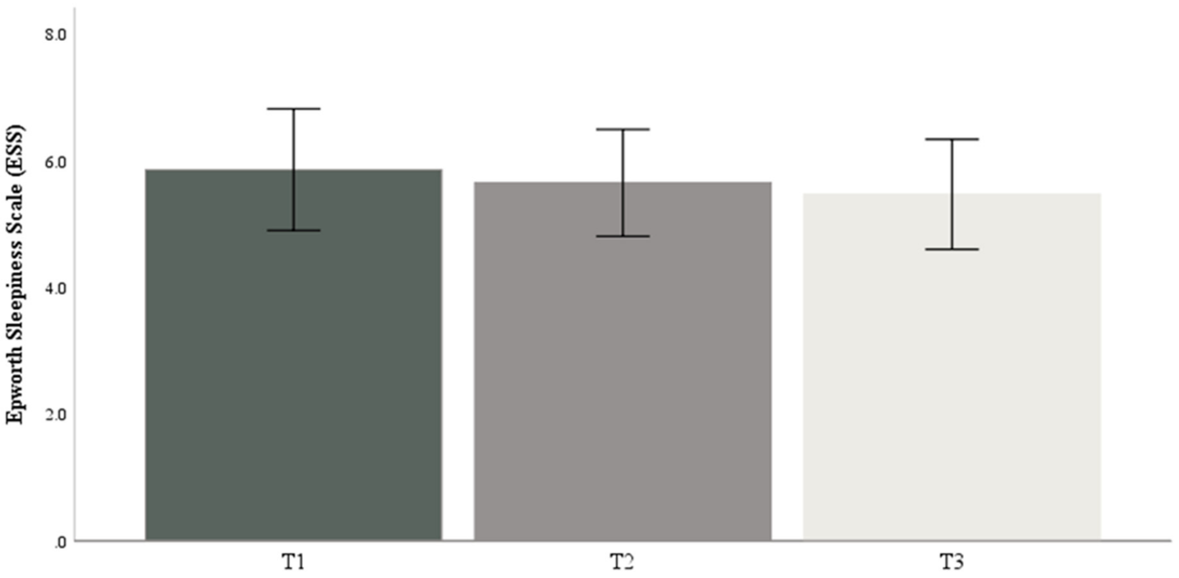


**Figure S9.** Mean Epworth Sleepiness Scale score for the three time points

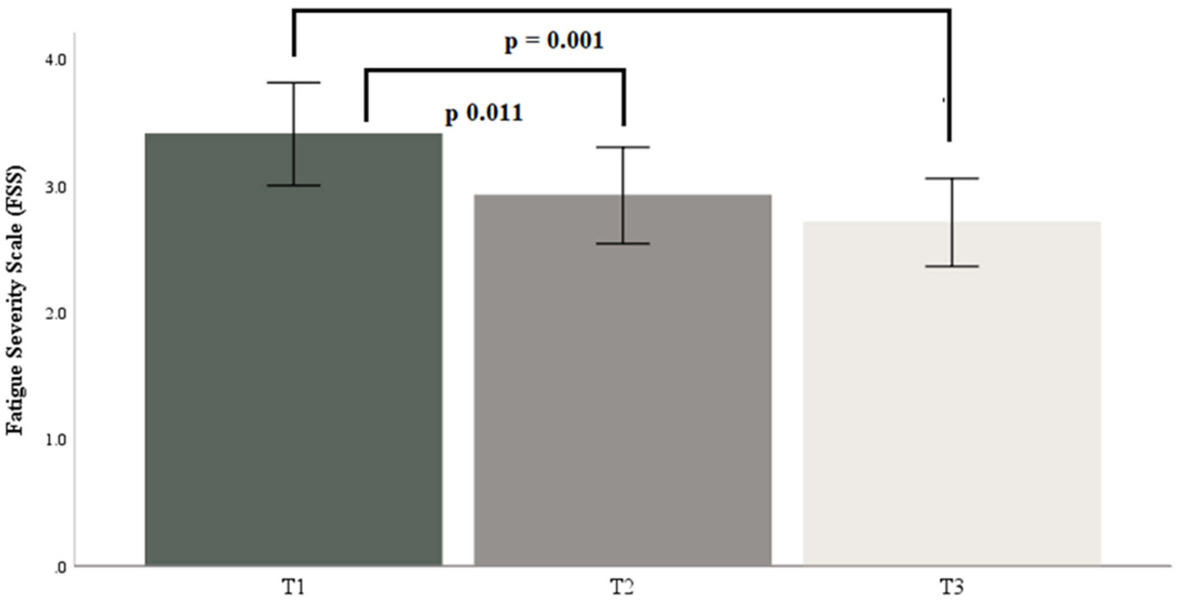


**Figure S10.** Mean Fatigue Severity Scale score for the three time points

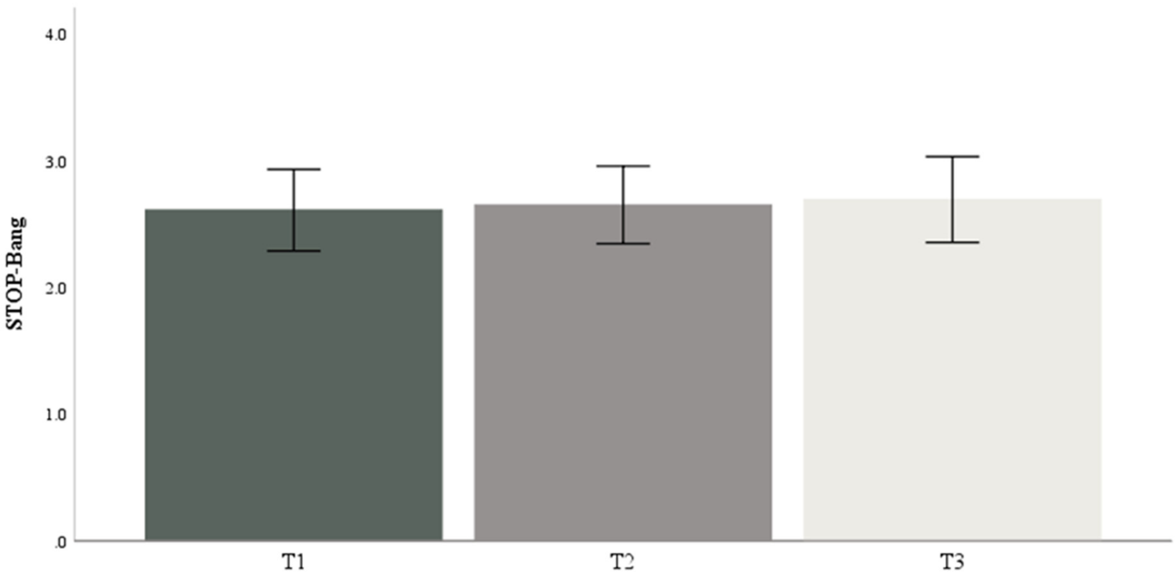


**Figure S11.** Mean STOP-Bang score for the three time points

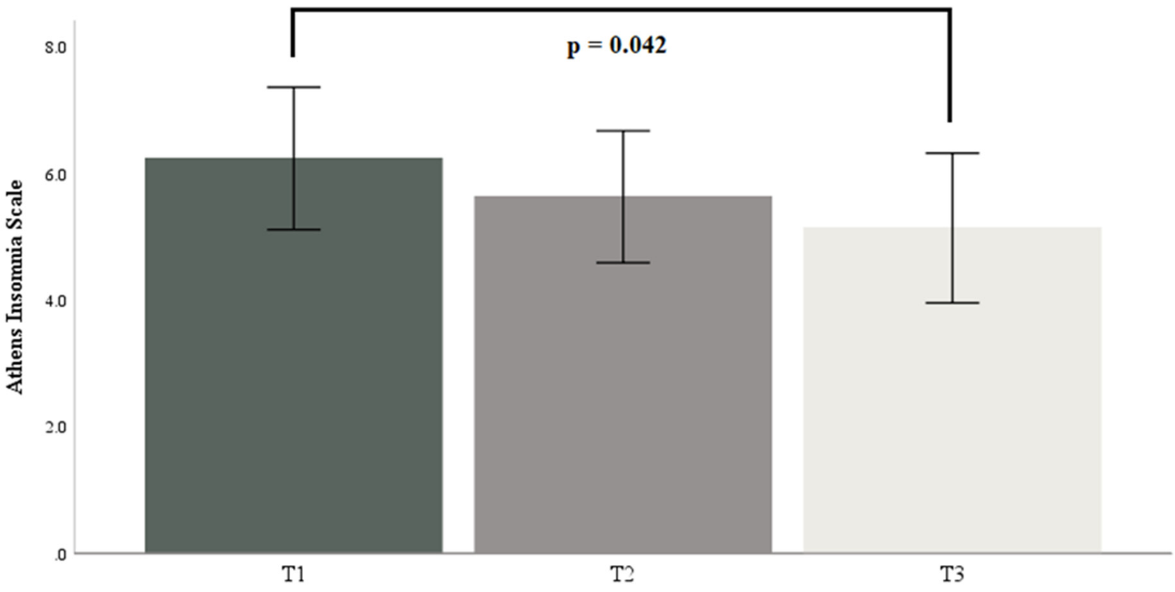


**Figure S12.** Mean Athens Insomnia Scale score for the three time points
